# Safety Considerations and Knowledge Gaps in Maternal Cardiopulmonary Resuscitation: A Systematic Review of Healthcare Professionals' Preparedness

**DOI:** 10.7759/cureus.89683

**Published:** 2025-08-09

**Authors:** Angeliki Sarella, Panagiota Tzela, Paraskevi Giaxi, Dimitra Sarantou, Anastasia Bothou, Kleanthi Gourounti

**Affiliations:** 1 Midwifery Department, Faculty of Health and Caring Sciences, University of West Attica, Athens, GRC; 2 Midwifery Department, School of Health and Care Sciences, University of West Attica, Athens, GRC; 3 Midwifery Department, University of West Attica, Athens, GRC; 4 Midwifery Department, General Hospital of Messinia – Nursing Unit of Kyparissia, Kyparissia, GRC

**Keywords:** cardiac arrest, cpr training, healthcare professionals, maternal cardiopulmonary resuscitation, medical education, obstetric emergency, perimortem cesarean, pregnancy, safety considerations, simulation training

## Abstract

Cardiopulmonary resuscitation (CPR) is a critical, life-saving intervention. In pregnant women, unique anatomical and physiological changes require adaptations to standard CPR protocols to ensure optimal outcomes for both mother and fetus, emphasizing the need for universal awareness and standardized training across diverse healthcare systems globally. Despite the high-risk nature of maternal cardiac arrest, evidence suggests that many healthcare professionals may not be adequately prepared to respond effectively. This systematic review followed PRISMA guidelines and included studies published between 2004 and 2024 identified through PubMed, Scopus, Web of Science, and Google Scholar. Boolean operators were used to combine keywords such as “cardiopulmonary resuscitation,” “pregnancy,” “knowledge,” and “training.” Studies assessing healthcare professionals’ knowledge or training in maternal CPR were eligible, and seven cross-sectional studies conducted in hospital-based settings met the inclusion criteria. Findings across all studies revealed a consistent gap in knowledge among healthcare providers, including anesthesiologists, obstetricians, emergency physicians, midwives, and nurses. Common deficits included awareness of CPR modifications specific to pregnancy, drug dosing, maternal positioning, and perimortem cesarean protocols. Previous specialized training or real-world clinical exposure positively influenced knowledge levels. The results highlight the urgent need not only for standardized maternal CPR guidelines, but also for their global implementation through mandatory continuing education and simulation-based training. This approach aims to improve preparedness and clinical competence, ultimately enhancing outcomes for both mothers and newborns during cardiopulmonary emergencies.

## Introduction and background

Cardiopulmonary resuscitation (CPR) during pregnancy is a rare but complex emergency requiring immediate, context-specific intervention. Maternal cardiac arrest presents a unique dual-patient challenge, though the primary aim of maternal CPR is to save the life of the mother, as fetal survival depends on maternal resuscitation. Cardiac arrest occurs in roughly 1 of every 9000 hospital deliveries (2017-2019), compared to 1 in 12,000 previously, with about 69% of affected women surviving to discharge [1]. Due to profound physiological and anatomical changes in pregnancy, such as increased blood volume, decreased functional residual capacity, and aortocaval compression by the gravid uterus, standard CPR protocols must be adapted to ensure optimal outcomes [2].

These modifications include manual left uterine displacement, lateral tilt positioning, early airway management, and, when indicated, perimortem cesarean delivery (PMCD). PMCD is clinically appropriate in pregnancies beyond 20 weeks’ gestation, when the uterus is palpable above the umbilicus. According to the Rοyal College of Obstetricians and Gynecologists (RCOG) Green-Top Guideline No. 56, if return of spontaneous circulation is not achieved within 4 minutes of cardiac arrest, PMCD should be initiated with the goal of fetal delivery by the fifth minute [3,4]. International guidelines from the Royal College of Obstetricians and Gynecologists, European Resuscitation Council, and the American Heart Association emphasize these steps; however, their clinical application remains inconsistent [4-6].

Multiple studies report that healthcare providers, including obstetricians, anesthesiologists, emergency physicians, nurses, and midwives, often lack sufficient training or awareness of maternal CPR protocols [7-9]. Factors such as clinical role, experience, and access to structured education contribute to varying levels of preparedness [10].

Despite the critical nature of maternal CPR, no prior systematic review has comprehensively evaluated healthcare professionals’ knowledge, training, and perceived competence in this area. This review aims to synthesize the existing evidence, identify key knowledge gaps, and propose targeted strategies to enhance preparedness through education, simulation, and standardized protocols.

## Review

Methods

Search Strategy and Literature Review

To identify relevant literature, a systematic search was conducted across four major databases: MEDLINE, Scopus, Web of Science, and Google Scholar. The search targeted peer-reviewed articles examining the knowledge, training, and preparedness of healthcare professionals involved in CPR in pregnant women. A predefined set of keywords was used, including “cardiopulmonary resuscitation,” “pregnancy,” “CPR,” “pregnant women,” “knowledge,” “education,” and “training.” Boolean logic (AND/OR) was applied to refine the strategy, using the combined query: (“cardiopulmonary resuscitation” OR “CPR”) AND (“pregnant women” OR “pregnancy”) AND (“knowledge” OR “training” OR “education”). The search strategy and article selection process were carried out in accordance with the PRISMA 2020 guidelines to ensure methodological transparency and consistency [11].

Inclusion Criteria

To be included in the review, studies had to meet several eligibility conditions. Only original research articles were accepted, with publication dates restricted to the last two decades (2004-2024). All studies had to be written in English and offer full-text availability in open-access format, allowing unrestricted use by students, researchers, and academics. Importantly, studies were included only if they focused specifically on pregnant women.

Exclusion Criteria

Non-original publications such as study protocols, books, essays, single-case reports, narrative reviews, letters to the editor, and meta-analyses were excluded from the review. Additionally, articles that were not accessible in full text through open-access platforms were excluded at the screening stage.

Identification and Screening Process

The identification phase initially retrieved a total of 22,560 publications - 92 from MEDLINE, 378 from Scopus, 217 from Web of Science, and 21,873 from Google Scholar. After applying the inclusion and exclusion criteria, 19,257 studies were eliminated: 59 from MEDLINE, 196 from Scopus, 154 from Web of Science, and 18,848 from Google Scholar. This left 3,303 articles for further evaluation. During the screening stage, 2,500 duplicate records were removed. Subsequently, 470 records were excluded based on title review, and another 200 were excluded after reading the abstracts or full texts.

Final Selection

**Figure 1:**
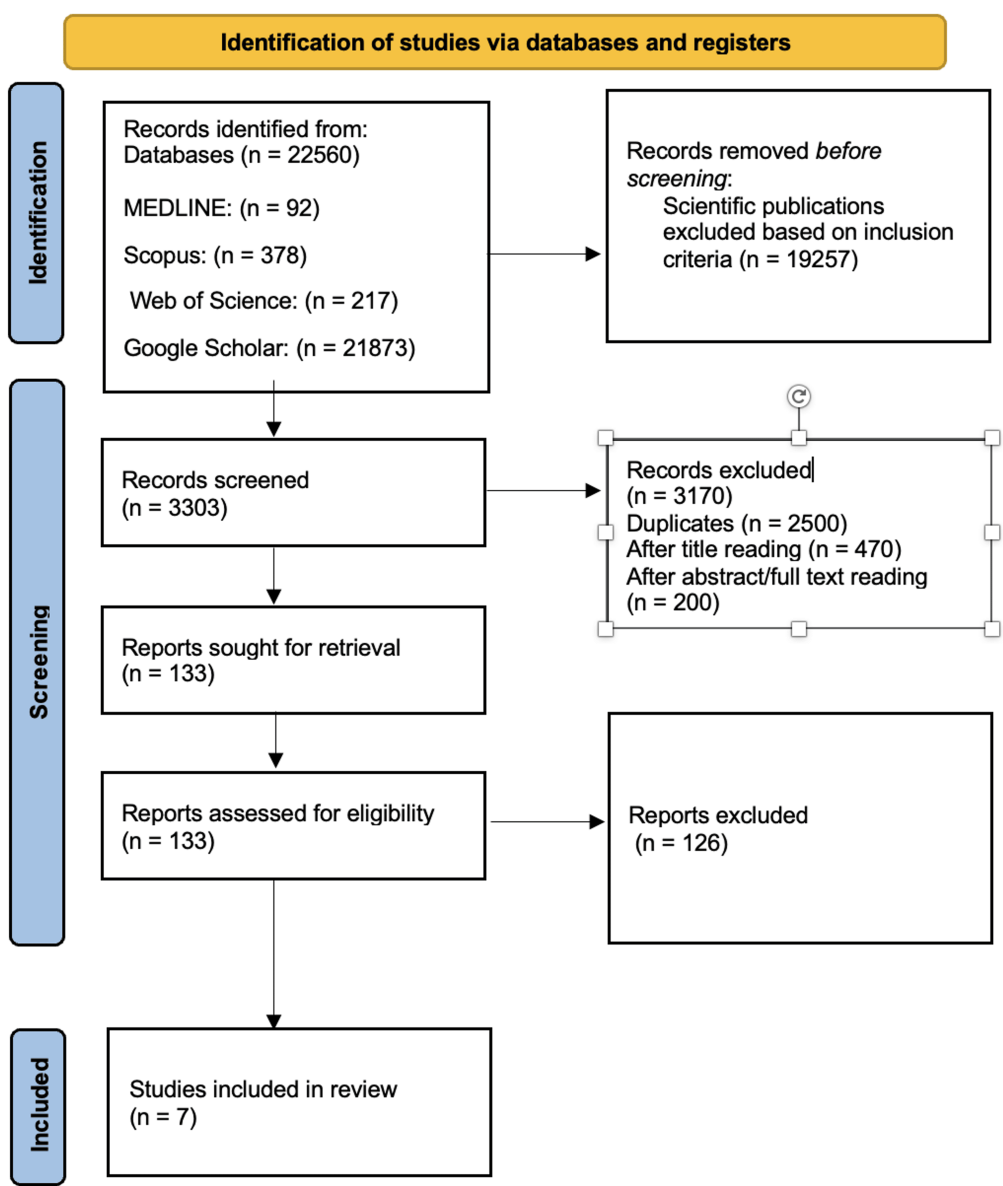
Selection process of selection studies Source: Ref. [11].

In the final eligibility assessment, 133 full-text studies were reviewed. Of these, 126 were excluded based on the predefined criteria, resulting in seven studies deemed suitable for inclusion in the present review. These studies form the basis of the evidence synthesis in the following section. The study selection process is summarized in Figure 1 using a PRISMA flow diagram.

*Data Extraction* 

All authors contributed to the process of data extraction and synthesis. Two authors independently performed the study selection and data extraction. Any discrepancies were resolved through consensus discussion. A third reviewer was available to adjudicate unresolved disagreements, though this was not necessary.

Key study characteristics were systematically compiled, including publication year, location, participant demographics (gender, age, profession), level of education, clinical experience, study setting, and methodological approach. The extracted data focused on how knowledge regarding maternal CPR was assessed, including the number of CPR-related questions, reasons for CPR, and summarized results. These details are presented in Table 1 and were used to guide the narrative synthesis and thematic discussion of findings.

**Table 1:** Summary of included studies CPR: cardiopulmonary resuscitation; ACLS: advanced cardiac life support.

Author	Title	Year of Publication	Number and Type of Healthcare Personnel Studied	Location and Country of Healthcare Personnel	Gender of Healthcare Personnel	Age of Healthcare Personnel	Educational Level/Experience of Healthcare Personnel	Method Used to Assess Knowledge on CPR in Pregnant Women	Number of CPR-Related Questions Asked	Reason for Performing CPR in Pregnant Women	Results
Mohamed et al. [12]	Maternity nurses’ performance regarding cardiopulmonary resuscitation during pregnancy: Simulation-based intervention.	2018	52 maternity nurses	Woman’s Health Nursing Department, Benha University Hospital, Egypt	Female (100 %)	Mean age 28.23 ± 5.02 years (52% between 20 and 29 years)	54% had secondary nursing education; 38% had <5 years of experience; mean experience 10.58 ± 6.35 years	Structured self-administered questionnaire and observational checklist assessing knowledge and performance, with simulation-based training intervention	16 knowledge questions, 59 total correct answers possible	Cardiac arrest	Significant improvement post-intervention in both knowledge and practice (p<0.001). Prior CPR training, education level, and experience were positively correlated with better post-test results (p<0.001).
Cohen et al. [13]	Assessment of knowledge regarding cardiopulmonary resuscitation of pregnant women	2008	75 participants: 34% anesthesiologists, 37% obstetricians, 20% emergency physicians (100% valid responses)	Stanford University Medical Center/Lucile Packard Children's Hospital, Stanford, California, USA	Not specified	Not specified	52% had received ACLS training within past 2 years, 20% within past 5 years; remainder had no training	One-hour questionnaire on left uterine displacement, ACLS algorithms, physiological changes in pregnancy, and perimortem cesarean recommendations	12	Cardiac arrest	Anesthesiologists scored higher than obstetricians (p=0.005) and emergency physicians (p<0.05) on ACLS and pregnancy physiology. No significant differences in left uterine displacement or cesarean recommendations. Emergency physicians performed best on ACLS items. Only 15% scored >85%, with average score 71±16%. No significant differences based on training history or visitor vs. local physicians.
Sathianathan et al. [14]	Effectiveness of simulation based teaching on knowledge and skill regarding cardiopulmonary resuscitation of pregnant women among nursing personnel	2020	50 nurses	Department of Obstetrics & Gynecology, Tertiary Care Center, Vellore, India	Female (100%)	Mean age 30.6 ± 6.8 years	Mean experience 6.1 ± 4.9 years; 16% held a nursing degree; 50% had attended >2 CPR-related courses	Questionnaire based on American Academy of Physicians’ obstetric advanced life support guidelines; scoring: 1 for correct, 0 for incorrect answers	20	Not specified	Pre-training scores: knowledge 8.14 ± 1.40, skills 10.62 ± 4.50. Post-training scores: knowledge 13.9 ± 2.3, skills 26.50 ± 2.2. Improvements were independent of age and experience, but post-training there was slight positive correlation with both.
Einav et al. [15]	A survey of labour ward clinicians’ knowledge of maternal cardiac arrest and resuscitation	2008	30 valid responses: 13 anesthesiologists, 13 obstetricians, 4 midwives	1st Congress on Obstetric and Perinatal Anesthesia, Israel – clinicians from 17 hospitals	Male (60%), female (40%)	Mean age 47 ± 9 years	43% had extensive experience, 50% moderate CPR experience; Israeli anesthesiologists are routinely trained at least once in advanced life support	Case-based questionnaire involving a pregnant woman deteriorating into cardiac arrest despite treatment	9	Cardiac arrest	Obstetricians were more likely than anesthesiologists to state that cricoid pressure was unnecessary during mask ventilation (61.5% vs. 15.4%, p = 0.027). No other significant differences were found between specialties. The study revealed inconsistent knowledge and unfamiliarity with resuscitation guidelines, with major deficits in basics (positioning, ventilation, defibrillation, drug dosing).
Grzeskowiak et al. [16]	Management of pregnancy-related emergencies: What do Polish anesthesiologists know?	2013	280 physicians: 10% residents, 46.4% medical graduates, 43.6% anesthesiologists	Two anesthesiology conferences in Popowo and Poznań, Poland	Female (58.9%), male (40%), 2 unspecified	55.7% aged 24-30; 16.1% aged 41-50; 14% aged 51-60; 11.4% aged 31-40	8.2% were department heads; 19% were consultants/senior assistants; 10% residents; 46.4% recent medical graduates	Bloom’s and Niemierko’s taxonomy-based questionnaire testing increasing cognitive levels—knowledge retention, application in emergencies, and assessment of basic and advanced knowledge regarding pregnancy-related emergencies and cardiac arrest. Compared to medical graduate control group.	9	Cardiac arrest, amniotic fluid embolism, eclampsia	57.5% claimed to have knowledge, 2.1% said they had insufficient knowledge, 20.7% said they had none. Among residents, 42.3% claimed partial knowledge on choking maneuvers and CPR cycles, yet performed poorly especially on cesarean timing questions. Basic knowledge overall was poor; residents outperformed anesthesiologists and graduates. Older anesthesiologists lacked basic handling knowledge but knew advanced management better.
Hardeland et al. [17]	Healthcare personnel self‐assessed competence and knowledge following implementation of a new guideline on maternal resuscitation in Norway. A repeated-measure study	2023	527 healthcare workers involved in CPR in pregnant women (251 in first survey, 182 in second), including physicians, anesthesiologists, gynecologists, obstetricians, midwives, nurses, specialized nurses, and nurse assistants	County hospital in Norway	Not specified	Not specified	Inclusion required >50% clinical work; mean clinical experience 17 years; 72% had CPR course participation, 10% had real-life CPR in pregnant women experience, 78% reported needing more advanced knowledge, 82% desired more training	Questionnaire covering demographics, training, self-assessed skills, roles and responsibilities, and theoretical knowledge on CPR and perimortem cesarean; second questionnaire assessed implementation and knowledge of new national maternal resuscitation guidelines	31	Not specified	Before guideline implementation: 72% reported general emergency knowledge, 48% good knowledge on maternal positioning, 62% airway handling, 71% poor on pre-delivery drug administration, similar post-delivery, 73% little knowledge on perimortem cesarean, 45% good on defibrillation. Half reported low competence in both CPR and perimortem cesarean. After guideline rollout: modest improvements (7-10%) in maternal positioning, drug administration, perimortem cesarean protocol, and CPR competence; slight decrease in defibrillation knowledge.
Kivungi [18]	A survey on knowledge, attitude and practice regarding cardiopulmonary resuscitation of pregnant women among anesthesiology and obstetrics registrars in Kenyatta National Hospital	2012	85 participants: 26 anesthesiologists and 59 obstetricians	Kenyatta National Hospital, Department of Obstetrics & Gynecology and Anesthesiology, Nairobi, Kenya	Male (59%), female (41%)	Mean age 31 ± 7 years (range 27-40)	52.5% had received CPR training; most trained >5 years prior; only 8 obstetricians had training on CPR in pregnancy; only 2.5% of participants reported full (100%) knowledge of obstetric CPR, while 48.7% reported 75% knowledge and 45% reported 50% knowledge	Paper questionnaire assessing knowledge, attitudes, and practices regarding CPR in pregnancy among hospital departments	14	Not specified	16.5% believed CPR may be unnecessary in some cases. Major reported challenges: collaboration (55%), medication (48%), knowledge (65%), equipment (77.5%). 97.5% were unaware of value of perimortem cesarean during CPR; 65.5% chose arrest location as ideal for cesarean. Nearly all emphasized help-seeking, documentation, positioning, and chest compressions. 91.3% of anesthesiologists used same defibrillation as non-pregnant; 40.5% of obstetricians used less energy. 71.25% reported epinephrine use in ACLS. Only 6 anesthesiologists and 1 obstetrician knew correct positioning; 90.91% knew about physiological changes in pregnancy and related CPR challenges.

Risk-of-Bias Assessment Tool 

The risk of bias in the included non-randomized studies was assessed using the Cochrane ROBINS-I tool (Risk Of Bias In Non-randomized Studies of Interventions). This tool evaluates bias across seven domains: confounding, selection of participants, classification of interventions, deviations from intended interventions, missing data, measurement of outcomes, and selection of the reported result, and the assessments are summarized in Table 2. Each study was independently evaluated, and any discrepancies were resolved through discussion. Studies identified as having “serious” or “critical” risk of bias in one or more domains were retained in the synthesis, with appropriate caution applied in the interpretation of findings, in accordance with current standards for systematic reviews.

**Table 2:** Risk-of-bias assessment using the ROBINS-I tool ROBINS-I: Risk Of Bias In Non-randomized Studies of Interventions.

Author, Year	Bias Due to Confounding	Bias in Selection of Participants	Bias in Classification of Interventions	Bias Due to Deviations From Intended Interventions	Bias Due to Missing Data	Bias in Measurement of Outcomes	Bias in Selection of the Reported Result	Overall Risk of Bias
Mohamed et al. 2018 [12]	Moderate	Low	Low	Low	Low	Moderate	Low	Moderate
Cohen et al. 2008 [13]	Moderate	Moderate to serious	Low	Not applicable	Low	Moderate	Low	Moderate
Sathianathan et al. 2020 [14]	Serious	Moderate	Low	Low	Low	Moderate	Low	Serious
Einav et al. 2008 [15]	Serious	Moderate	Low	Not applicable	Low	Moderate	Low	Serious
Grzeskowiak et al. 2013 [16]	Serious	Moderate	Low	Not applicable	Low	Moderate	Moderate	Serious
Hardeland et al. 2023 [17]	Moderate	Moderate	Low	Moderate	Moderate	Low	Low	Moderate
Kivungi 2012 [18]	Moderate	Moderate	Not applicable	Not applicable	Low	Moderate	Low	Moderate

Synthesis of Findings

Due to significant heterogeneity in study design, population characteristics, outcome measures, and data collection instruments across the included studies, a meta-analysis was not feasible. As such, a narrative synthesis approach was adopted, consistent with PRISMA and Cochrane recommendations for reviews with diverse methodologies and non-comparable quantitative outcomes. This method allowed for a structured, qualitative integration of findings, organized thematically to highlight patterns in knowledge levels, training efficacy, professional roles, and regional disparities. Although no pooled statistical measures (e.g., confidence intervals or p-values) were calculated, the synthesis was grounded in rigorous comparative analysis of study outcomes, with bias assessments informing the weighting of conclusions. A statistical reviewer was not required given the qualitative nature of the data and the absence of effect size estimation or meta-regression.

Results 

This systematic review evaluated seven cross-sectional studies that investigated the level of knowledge, competence, and preparedness of healthcare professionals in the context of CPR during pregnancy. In total, 1,099 healthcare professionals were assessed across these studies, spanning international healthcare settings, including Egypt, the United States, India, Israel, Poland, Norway, and Kenya, and encompassing a wide array of professional roles such as anesthesiologists, obstetricians, emergency physicians, midwives, and nurses.

The study by Mohamed et al. [12] evaluated 52 maternity nurses in Egypt and employed a structured simulation-based educational intervention. A statistically significant increase in both knowledge and clinical performance scores was observed post-intervention (p < 0.001), indicating that targeted simulation training can markedly improve proficiency. Notably, participants with prior CPR training, higher educational qualifications, and more years of clinical experience demonstrated superior post-test outcomes.

In Cohen et al. [13], a cross-sectional survey conducted at Stanford University Medical Center (USA) assessed 75 physicians (34% anesthesiologists, 37% obstetricians, 20% emergency physicians). Despite many having received prior advanced cardiac life support (ACLS) training, only 15% of participants achieved a score above 85%, with an overall average of 71% (±16%). While anesthesiologists scored significantly higher than their obstetric and emergency physician counterparts in questions related to physiological adaptations in pregnancy and ACLS algorithms (p < 0.05), considerable gaps persisted in critical domains such as left uterine displacement and PMCD.

Sathianathan et al. [14] investigated the impact of simulation-based training among 50 nurses in India. Pre- and post-training evaluations revealed significant improvements in knowledge (mean score increased from 8.14 ± 1.40 to 13.9 ± 2.3) and skills (from 10.62 ± 4.50 to 26.5 ± 2.2). Interestingly, while training benefits were universal across demographic strata, weak positive correlations emerged between knowledge gains and participants’ age and professional experience.

Einav et al. [15] conducted a case-based assessment among 30 healthcare professionals (13 obstetricians, 13 anesthesiologists, and four midwives) from 17 Israeli hospitals. The results demonstrated a heterogeneous understanding of maternal CPR guidelines. A statistically significant divergence was identified in perceptions regarding the necessity of cricoid pressure during ventilation, with obstetricians more likely than anesthesiologists to consider it unnecessary (61.5% vs. 15.4%, p = 0.027). Across the sample, knowledge deficits were observed in fundamental CPR procedures, including patient positioning, defibrillation techniques, and drug administration.

The Polish study by Grzeskowiak et al. [16], which assessed 280 physicians, revealed that although the majority perceived themselves as knowledgeable, objective evaluation suggested poor foundational knowledge, particularly among older practitioners. Remarkably, recent medical graduates and residents outperformed more senior professionals in baseline knowledge, underscoring potential discrepancies in initial versus continuing education.

Hardeland et al. [17] conducted a two-phase survey before and after the implementation of updated maternal resuscitation guidelines in Norway. Among 527 healthcare workers, modest knowledge improvements (7-10%) were observed in post-intervention assessments on maternal positioning, drug administration, and PMCD protocols. Nevertheless, a substantial proportion (78%) reported ongoing insufficiencies in knowledge and expressed a strong need for advanced training, highlighting the limited impact of passive dissemination of guidelines alone.

Finally, Kivungi [18] reported findings from 85 anesthesiology and obstetrics registrars in Kenya. The study revealed severe deficiencies in both theoretical knowledge and practical preparedness. Only 2.5% of respondents reported complete familiarity with maternal CPR protocols. Alarmingly, 97.5% were unaware of the importance of PMCD, and a significant portion demonstrated misconceptions about CPR drug dosages, defibrillation energy settings, and optimal positioning during resuscitation.

Collectively, these findings underscore a pervasive global deficiency in maternal CPR knowledge, transcending country income levels and professional categories. While simulation-based educational interventions demonstrated efficacy in improving knowledge and performance outcomes, the absence of standardized training curricula and inconsistent exposure to real-life obstetric emergencies contribute to persistent gaps. Moreover, the heterogeneity in assessment methodologies, sample characteristics, and outcome measures across studies limits generalizability but nonetheless strengthens the call for harmonized, interdisciplinary training programs with periodic evaluation of competence.

Discussion 

This systematic review highlights the persistent knowledge gap among healthcare professionals in managing CPR during pregnancy, a clinical scenario that remains both rare and highly complex. Across all seven reviewed studies, significant variability in awareness, confidence, and practical preparedness was evident among diverse professional groups, including obstetricians, anesthesiologists, emergency physicians, midwives, and nurses [12-18].

Notably, studies such as Mohamed et al. [12] and Sathianathan et al. [14] demonstrated substantial improvements in both knowledge and skills following simulation-based interventions. These results emphasize the effectiveness of experiential learning modalities in addressing deficits among nursing staff. Similarly, Hardeland et al. [17] showed modest gains in competence after the implementation of national guidelines, though baseline knowledge of critical topics such as perimortem cesarean section remained low.

In physician populations, Cohen et al. [13] and Einav et al. [15] identified significant gaps in familiarity with pregnancy-specific modifications to CPR, particularly among emergency physicians and obstetricians, even among those who had completed general ACLS training. However, as ACLS is not designed for obstetric emergencies, this highlights the need for wider implementation of obstetric-specific training programs such as Advanced Life Support in Obstetrics (ALSO) [19]. These findings are reinforced by the work of Lipman et al. [20], who documented inadequate preparedness across US labor wards, underscoring a systemic deficit even in high-resource settings.

Interestingly, exposure to real-life maternal arrest scenarios or recent targeted training was consistently associated with improved performance [13,16,17]. This supports evidence from broader literature indicating that practical, context-specific training enhances retention and response accuracy. For instance, Baldi et al. [21] reported wide variability in final-year medical students’ CPR competence, underscoring that even at the training stage, exposure and repetition significantly influence performance.

The reviewed literature also points to structural and systemic issues. Kivungi [18] and Grzeskowiak et al. [16] both reported that institutional factors, such as lack of access to updated protocols or inconsistent national guidelines, contribute to professional uncertainty. Furthermore, several studies relied on self-reported confidence levels or knowledge questionnaires rather than observed performance under clinical pressure, which limits the real-world applicability of findings. This issue is mirrored in the broader literature, including in studies such as those by Draycott et al. [22], which stress the importance of high-fidelity simulations and multidisciplinary team-based training for real improvement in clinical outcomes.

The generalizability of the present findings is inherently constrained by marked heterogeneity in study design, participant composition, and the cultural and structural contexts of healthcare delivery. Notably, included studies spanned both high- and low-income countries, such as Norway [17], Kenya [18], Egypt [12], and India [14], each with distinct healthcare infrastructures and training frameworks. For instance, the study by Hardeland et al. [17] encompassed a heterogeneous cohort of healthcare personnel, including physicians, midwives, nurses, and auxiliary staff. This broad inclusion complicates the extrapolation of results to specific professional categories, as key interventions during maternal cardiac arrest, such as PMCD and airway management, are predominantly within the purview of obstetricians and anesthesiologists. Consequently, conclusions regarding competence or preparedness cannot be uniformly applied across all provider types.

Moreover, while healthcare systems in high-income countries may benefit from greater access to structured training programs, simulation-based education, and standardized emergency protocols, evidence from the literature reveals that critical knowledge gaps in maternal CPR persist even in well-resourced settings. The consistency of these deficits across geographically and economically diverse contexts underscores the existence of a global challenge in ensuring adequate maternal resuscitation preparedness. Such a challenge is compounded by variability in training exposure, clinical responsibilities, and institutional readiness, necessitating a concerted, system-wide approach to educational reform and professional development.

The importance of standardized, evidence-based training has been repeatedly underscored by the American Heart Association (AHA), particularly in their scientific statement on maternal cardiac arrest [23], which outlines critical alterations in positioning, pharmacologic dosing, and the timing of perimortem cesarean section. Nevertheless, studies such as Jeejeebhoy et al. [24] show that these recommendations remain poorly disseminated in many healthcare settings.

There is also a paucity of longitudinal studies exploring the retention of maternal CPR skills over time. Most available research utilizes cross-sectional designs, limiting conclusions about the lasting impact of interventions. Future studies should examine long-term competence, assess actual clinical performance, and evaluate whether improvements translate into better maternal and neonatal outcomes.

In summary, despite the low incidence of maternal cardiac arrest, the risk it poses necessitates a heightened level of preparedness. Multidisciplinary, scenario-based education, aligned with current guidelines and routinely reinforced, is essential. Institutions should also adopt mandatory training modules with periodic re-certification, as proposed by Fransen et al. [25], to maintain competency and ensure readiness in high-stakes obstetric emergencies.

Limitations and recommendations

This review has several limitations. First, most of the included studies were cross-sectional and relied on self-reported questionnaires, which may overestimate actual competence. Second, there was considerable heterogeneity in study populations, methods, and geographic settings, which limits generalizability. Third, few studies evaluated long-term retention of CPR skills or actual clinical performance under emergency conditions. Additionally, several studies presented moderate to serious risk of bias, particularly in domains such as confounding, participant selection, and outcome measurement. For example, studies by Sathianathan et al. [14], Einav et al. [15], and Grzeskowiak et al. [16] demonstrated serious risk of bias in multiple domains, including confounding and selection processes. Although no study was excluded based on methodological limitations, findings from higher-risk studies were interpreted with appropriate caution.

Future research should prioritize longitudinal study designs, objective performance assessments, and exploration of training efficacy across different healthcare systems. Furthermore, there is a pressing need for the development and adoption of a unified international competency framework for maternal CPR. Such a standard would ensure consistency in provider training, improve clinical preparedness globally, and reduce variability in outcomes, particularly in high-stakes obstetric emergencies. Healthcare institutions should also integrate standardized, scenario-based maternal resuscitation training as a mandatory component of professional development.

## Conclusions

This systematic review highlights significant knowledge gaps and training deficiencies among healthcare professionals in managing CPR during pregnancy. Despite being a rare event, maternal cardiac arrest poses a critical threat to both maternal and fetal survival, requiring rapid, well-coordinated interventions. The evidence suggests that many clinicians, including anesthesiologists, obstetricians, midwives, nurses, and emergency personnel, lack adequate preparation in this domain, particularly in essential skills such as uterine displacement, defibrillation, pharmacologic considerations, and PMCD.

There is a pressing need to improve safety and clinical competence in maternal CPR through the development and implementation of standardized, evidence-based guidelines. Additionally, mandatory continuing education and high-fidelity simulation-based training programs should be prioritized to strengthen healthcare professionals’ preparedness. Addressing this gap is not only critical for enhancing clinical outcomes but also an ethical imperative to reduce preventable maternal and neonatal mortality in cardiopulmonary emergencies. As such, ensuring adequate training in maternal CPR is both a global health priority and a shared ethical responsibility across healthcare systems.
